# The Genetic Mechanisms and Pathology of Atrial Fibrillation: A Narrative Review

**DOI:** 10.3390/biomedicines13030654

**Published:** 2025-03-07

**Authors:** Elio Zito, Lorenzo Bianchini, Elena Sommariva, Mariabeatrice Costa, Giovanni B. Forleo, Claudio Tondo, Marco Schiavone

**Affiliations:** 1 Cardiology School, University of Milan, 20122 Milan, Italymbea.costa97@gmail.com (M.C.); 2 Department of Clinical Electrophysiology & Cardiac Pacing, Centro Cardiologico Monzino, IRCCS, 20138 Milan, Italyclaudio.tondo@cardiologicomonzino.it (C.T.); 3 Unit of Inherited Cardiomyopathies, Centro Cardiologico Monzino, IRCCS, 20138 Milan, Italy; elena.sommariva@cardiologicomonzino.it; 4 Cardiology Unit, Luigi Sacco University Hospital, 20157 Milan, Italy; forleo@me.com; 5 Department of Biomedical, Surgical and Dental Sciences, University of Milan, 20122 Milan, Italy

**Keywords:** atrial fibrillation, genetic, pathophysiology, triggers

## Abstract

Atrial fibrillation (AF), the most prevalent tachyarrhythmia worldwide, is a complex condition influenced by genetic, structural, and environmental factors. While AF in the elderly is often associated with underlying cardiac disease, early-onset or “lone” AF (LAF) exhibits a stronger genetic predisposition. Studies have identified both monogenic and polygenic contributors to AF risk. Monogenic mutations, inherited in Mendelian patterns, often affect ion channels and regulatory proteins, while polygenic variants modulate susceptibility and interact with environmental factors. Genome-wide association studies (GWAS) and exosome-wide association studies (ExWAS) have expanded our understanding of AF genetics, identifying numerous susceptibility loci, though challenges remain in linking these variants to specific molecular mechanisms. Pathophysiologically, AF results from a balance of triggers, drivers, and substrates. Triggers, such as ectopic foci in the pulmonary veins, initiate AF episodes, while structural and electrical remodeling perpetuates the arrhythmia. Fibrosis, atrial dilation, and tachycardia-induced remodeling promote reentry circuits and irregular conduction, increasing AF vulnerability. The interplay between genetic predisposition and remodeling processes underscores the complexity of AF maintenance, particularly in persistent AF forms. Emerging insights into AF genetics and pathophysiology highlight the need for personalized approaches to its prevention and management. Understanding genetic risk, combined with targeted therapies addressing structural and electrical remodeling, holds promise for improved patient outcomes. Future research into AF’s molecular and genetic mechanisms will be key to advancing precision medicine in this field.

## 1. Introduction

Atrial fibrillation (AF) is the most commonly diagnosed clinical tachyarrhythmia, affecting 33 million people worldwide [1]. In the elderly, AF is often associated with underlying heart disease, such as ischemic, hypertensive, myocardial, or valvular disease. Interestingly, in 10% to 30% of patients with AF, no definitive structural defect or previous cardiac pathology is present. The term ‘lone AF’ (LAF) was first introduced in 1954 by Evans and Swann to describe AF patients for whom no apparent heart disease could be found during subsequent investigations [2]. Current guidelines define LAF as AF in younger adults (age < 60 years) with no clinical history or echocardiographic evidence of concomitant cardiovascular or pulmonary conditions or any acute trigger [3]. This term has also been used interchangeably to refer to patients suffering from early-onset AF and includes those affected by familial AF. The role of genetic predisposition in AF development has been explored by several studies: In the Framingham Offspring Study, it was found that those who had one parent with AF had a 1.8-fold increase in the risk of developing AF, with a three-fold higher risk for those under 75 years old [4]. In the Mayo Clinic AF registry, 5% of all patients and 15% of LAF patients had a family history of AF [5]. Similarly, in another study among 5000 Icelanders, first-degree relatives of AF patients were 1.77 times more at risk of developing AF than the general population [6]. This trend has also been confirmed in a Danish cohort, where the incidence rate for LAF was 3.48 in subjects with affected first-degree relatives and 1.64 in those with affected second-degree relatives [7].

Since the first documentation of idiopathic AF by Evans and Swann, there has been a significant expansion in the description of etiologic factors, alongside the identification of new forms of heart disease through modern imaging and testing methods (e.g., atrial fibrosis, diastolic dysfunction, heart failure with preserved ejection fraction, channelopathies). This progress has dramatically reduced reported LAF prevalence from approximately 30% to 5–10% of cases. For example, in a recent survey conducted by the European Heart Rhythm Association, the prevalence of LAF was reported to be as low as 10% [3]. Despite the variability in prevalence rates across different populations, the genetic background appears to play an incontrovertible role in the genesis and maintenance of AF. From the reported data, it is evident that heritability is more pronounced in juvenile/idiopathic forms of AF, though a genetic predisposition is still present in late-onset forms. Elucidating the molecular bases of LAF’s heritability patterns could potentially lead to the early identification of at-risk patients, the development of tailored clinical approaches, and advancements in prognostic and stratification tools. Studying the genetic contributors of AF in families with a history of genetic AF could also shed light on possible molecular mechanisms shared between early-onset and late-onset forms. This knowledge might be used to explain differences in patients’ responses to medical therapy and their predisposition to AF recurrence following catheter ablation (CA) in different AF subsets. The aim of our review is to present in a clear, concise, and complete way the latest acquired knowledge on AF genetics and pathophysiology, providing examples of genetic and pathophysiology interplay.

## 2. Genetic AF Background: Monogenic and Polygenic Contributors to AF Risk, Analysis Techniques, and Polygenic Risk Score

The mosaic puzzle of AF heritability comprises two types of genetic contributors, rare high-impact variants and common low-impact and low-penetrance variants. The former have been exclusively described in families with multiple members affected by the familial form of AF (genetic AF) and were discovered through linkage or segregation analyses, showing an autosomal dominant and in rare cases X-linked inheritance pattern (Table 1).

Most of the variants linked to LAF are associated with genes encoding ion channel proteins, which are believed to directly affect the action potential. The first pathogenic variants linked to familial AF were found in the ion channel gene *KCNQ1* by Chen et al., a gene that encodes the α subunit of the IKs current, also responsible for Long QT syndrome1 [8]. Since 2003, many studies reported gain-of-function (GOF) mutations in genes coding potassium channels. While most of the reported variants were GOF mutations, loss-of-function (LOF) mutations have also been identified (Table 1) [9]. Other GOF mutations have also been identified in *KCNE1*, *KCNE2*, *KCNE*, *KCNE5*, *KCNQ1*, and *KCNJ2* genes [10,11,12,13,14,15,16,17]. Also, mutations in SCN5A were reported to be related to AF as well as mutations in genes encoding the four regulatory b-subunits (*SCN1B*, *SCN2B*, *SCN3B*, *and SCN4B* [18,19,20]. *SCN10A* gene has also been described as potentially implicated in the pathogenesis of AF: it encodes the Na 1.8 sodium channels, which is believed to be responsible for late sodium currents, and mutations could prolong PR and QRS intervals [21]. Some groups also described mutations in genes encoding for proteins of intercellular ap junctions and connexosomes, most notably the cardiac connexin genes *GJA1* and *GJA5*, the genes encoding connexin 40 and connexin 43 [22,23]. Many mutations in transcription factor genes have also been reported to be related to AF (*NKX2-5*, *PITX2*, *GATA4*, *GATA5*, and *GATA6* genes) [24,25,26,27,28]. Other examples include the cardiac natriuretic peptide A gene, *NPPA*, and *GATA* and *PITX2* genes.

**Table 1:** A list of genes associated with LAF. Rare pathogenic variants have been found in the mentioned genes in correspondence with the indicated locus. The mode of inheritance is either autosomal dominant or X linked. The mechanism of action may be a loss of function or a gain of function. Adapted from Ahmed et al. [9].

Gene	Locus	Mode of Inheritance	Functional Effect
*KCNQ1*	11p15.5	Autosomal dominant	Gain of function
*KCNE1*	21q22.1	Autosomal dominant	Gain of function
*KCNE2*	21q22.1	Autosomal dominant	Gain of function
*KCNE3*	11q13.4	Autosomal dominant	Gain of function
*KCNE5*	Xq23	X-linked	Gain of function
*KCNJ2*	17Q23.1	Autosomal dominant	Gain of function
*KCNJ5*	11q24.3	Autosomal dominant	Gain of function
*KCNJ8*	12p12.1	Autosomal dominant	Gain of function
*KCNH2*	7q36.1	Autosomal dominant	Gain of function, Loss of function
*KCNA5*	12p13.32	Autosomal dominant	Gain of function, Loss of function
*KCND3*	1p13.2	Autosomal dominant	Gain of function
*HCN4*	15q24.1	Autosomal dominant	Loss of function
*MYH6*	14q11.2	Autosomal dominant	Loss of function
*ABCC9*	12p12.1	Autosomal dominant	Loss of function
*RYR2*	1q43	Autosomal dominant	Gain of function
*CACNB2*	10p12	Autosomal dominant	Loss of function
*CACNA2D4*	12p13.33	Autosomal dominant	Loss of function
*CAV1*	7q31.2	Autosomal dominant	Loss of function
*SCN1B*	19q13.11	Autosomal dominant	Gain of function, Loss of function
*SCN2B*	11q23.3	Autosomal dominant	Loss of function
*SCN3B*	11q24.1	Autosomal dominant	Loss of function
*SCN4B*	11q23.3	Autosomal dominant	Loss of function
*SCN5A*	3p22.2	Autosomal dominant	Gain of function, Loss of function
*SCN10A*	3p22.2	Autosomal dominant	Gain of function, Loss of function
*GATA4*	8p23.1	Autosomal dominant	Loss of function
*GATA5*	20q13.33	Autosomal dominant	Loss of function
*GATA6*	18q11.2	Autosomal dominant	Loss of function
*GJA1*	6q22.31	Autosomal dominant	Loss of function
*ZFHX3*	16q22.2-q22.3	Autosomal dominant	Loss of function
*GREM2*	1q43	Autosomal dominant	Gain of function
*JPH2*	20q13.12	Autosomal dominant	Loss of function
*LMNA*	1q22	Autosomal dominant	N/A
*NUP155*	5p13.2	Autosomal dominant	Loss of function
*SYNE2*	14q23.2	Autosomal dominant	N/A
*NKX2-5*	5q34	Autosomal dominant	Loss of function
*NKX2-6*	8p21.2	Autosomal dominant	Loss of function
*NPPA*	1p36.22	Autosomal dominant	Loss of function
*PITX2c*	4q25	Autosomal dominant	Loss of function

Recently, extensive genome comparison analyses between AF patients and controls in large population studies, such as genome-wide association studies (GWASs) and whole exome sequencing (WES), have facilitated the identification of novel candidate genes. Unlike the genetic mutations detected in familial AF, the variants identified in these studies tend to be more widely distributed among the general AF population and lack a strong genotype–phenotype correlation. This suggests that they can modulate individual susceptibility (even acting synergistically with other variants and/or environmental factors) to promote AF rather than directly causing it. This occurs in a polygenic or even complex-disease fashion, with each locus acting as a disease or susceptibility modifier. For these reasons, establishing a clear and statistically robust correlation with disease traits has proven technically challenging and less intuitive. A GWAS analyzes the association between specific genetic loci (tagged by single nucleotide polymorphisms, SNPs) and the disease. Each associated SNP could either be the actual variant producing the effect or be in linkage disequilibrium with the disease modifier. In addition, SNPs are often located in non-coding regions of the genome, so their pathogenic role can only be explained by presuming their ability to alter the activity of a transcriptional regulatory element, such as an enhancer or repressor, which in turn modifies the transcription of a nearby gene. In the first GWAS for AF, it was reported that carrying a single variant at the 4q25 locus near the gene *PITX2* conferred over a 60% increased risk of disease in the general population, with an even greater risk in younger individuals [29]. Large-scale genotype data from European and Japanese populations were recently analyzed, leading to the identification of 13 new common susceptibility genetic loci and an additional one unique to the Asian population (*PRRX1*, *CAV1*, *SYNE2*, *C9orf3*, *HCN4*, *SYNPO2L*, *ZFHX3*, *NEURL*, *GJA1*, *TBX5*, *CAND2*, and *CUX2*) [30,31,32].

Importantly, for most GWAS variants, there is usually no straightforward association with a specific gene or disease mechanism, as a single regulatory region could include several neighboring genes. Bridging the gap from variants to genes remains a major challenge in disease genetics, particularly for complex polygenic traits such as AF. Moreover, another major limitation of a GWAS is its ability to link disease traits only to known variations in the genome. In this regard, whole exome sequencing (WES) provides an interesting tool for assessing the genetic background of patients, given its ability to sequence the entire protein-coding genome at once, called the exome. For example, a meta-analysis of common and rare variant association studies, including 31 GWASs and 2 WES studies from the AFGen consortium, identified 12 additional genetic loci *(METTL11B/KIFAP3*, *ANXA4/GMCL1*, *CEP68*, *TTN/TTN-AS1*, *KCNN2*, *KLHL3/WNT8A/FAM13B*, *SLC35F1/PLN*, *ASAH1/PCM1*, *SH3PXD2A*, *KCNJ5*, *SLC35F1/PLN*, *SOX5*, and *SH3PXD2A*) [33].

Another potential solution to this problem is incorporating expression quantitative trait loci (eQTL) mapping. An eQTL analysis links the genotype of an SNP at an AF locus to the expression of genes in the region. If an AF-associated SNP is strongly linked to the expression of a single gene, that gene is likely to be the causative gene at the locus. The feasibility of this approach has been validated in several studies [34], and in a recent work, a reference list of gene expressions in human left atrial appendage tissues was established to serve as a control for future research [35]. To date, the most complete description of AF’s complex genetic architecture has been provided in three recent works by Roselli et al. and Nielsen et al., which identified up to 138 different genetic susceptibility loci [36,37,38] (Table 2).

**Figure 1:**
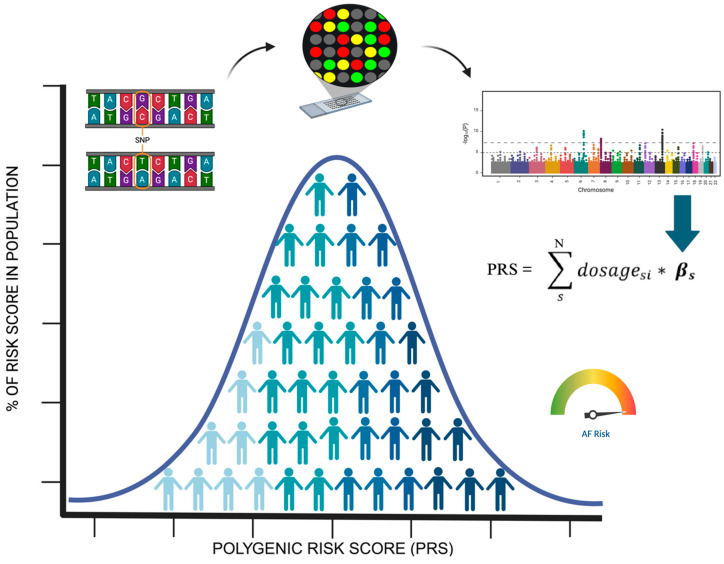
A PRS is calculated for each individual as a sum of the product of genetic dosage and a weight. The weights are derived from the effect estimates of a GWAS. The PRS of individuals in a population follows a gaussian distribution. Individuals in the highest percentile of the distribution show an increased risk for AF versus the remaining population. PRS: polygenic risk score; adapted from Roselli et al. [38].

Interestingly, the polygenic nature of AF, as captured in GWASs, can be utilized to create a genetic risk score, which is calculated for each individual. Since complex phenotypes may arise from the aggregate effects of thousands of variants, each with its own associated risk, polygenic risk scores (PRSs) are developed by weighting the contribution of each variant within an individual’s genome [38] (Figure 1).

Many PRSs have been developed for AF, with studies demonstrating significant associations between PRSs and AF risk. An initial AF PRS used the top 12 genome-wide significant genetic variants, while more contemporary risk calculator tools incorporate over 6.6 million variants [39]. In this latter approach, individuals in the top 1.5% with a high PRS had more than a four-fold increased risk of AF. In one interesting application of an AF PRS, Weng et al. [40] found that individuals in the highest tertile of polygenic risk score had a higher lifetime risk for AF (47%) compared with the individuals in the lowest tertile (26%). One influential study by Khera et al. developed a genome-wide PRS comprising 6,730,541 SNPs, demonstrating that those with a score in the top 1% of the cohort had almost five times the risk of AF than did the rest of the cohort [39]. Nevertheless, in the most recent guideline statements, genetic testing for AF is not recommended [41]. A recent consensus document regarding genetic testing for cardiac diseases from the EHRA, HRS, APHRS, and LAHRS suggested genetic testing in certain genes. Due to the observed association of AF with other phenotypes (Brugada, conduction disease, cardiomyopathy) or those that may be isolated, probably particularly in young individuals, genes encoding both ion channels and sarcomere genes may be considered, including cascade screening in the family members in case of pathogenic or likely pathogenic variants. These results can play an important role in the delineation of the prognosis of these patients [42]. However, emerging data suggest that diagnostic testing with targeted gene sequencing may have a role in evaluating patients with early-onset AF, especially for those without pre-existing cardiac disease (no ischemic heart disease or cardiomyopathies). In this patient population, choosing to start on physician-based decision routine cascade genetic testing could be useful to identify patients at a higher risk of incidental asymptomatic AF and thus higher stroke risk (family members of index patients affected by early-onset AF. On the contrary, the role of polygenic testing in older AF population remains more controversial: a PRS does not predict as well as monogenic testing one’s predisposition to AF development. Interestingly, several studies started to shed light on the potential applications of an SNP polymorphism analysis in AF patients’ cohorts: several authors reported SNPs ability to predict AF recurrence after catheter ablation and/or drug therapy response (flecainide potency and individualized therapeutical doses of antiarrhythmic drugs) [43,44,45,46]. The non-randomized and retrospective nature of the great part of these findings makes the PRSs and the SNPs still unsuitable for a large-scale clinical application. More data are needed to be collected before we could suggest PRS adoption in our routine AF management workflow.

A contemporary view of AF etiology posits that both genetic and environmental factors, as well as their interactions, contribute to disease development. Until the genetic basis of AF is better understood, widespread genetic testing may result in low diagnostic yield and the identification of false positives or variants of uncertain significance. Nonetheless, substantial evidence suggests that identifiable genetic variants play a critical role in mediating AF susceptibility in specific patient populations.

## 3. Pathophysiology of AF: Balance of Triggers, Drivers, and Substrate

The modern conception of AF assumes that both disease triggers and driver factors are necessary for AF to manifest, persist, and self-renew. Generally, patients with frequent, self-terminating episodes of AF are likely to have a predominance of triggering factors, whereas those with AF that do not terminate spontaneously are more likely to exhibit perpetuating factors. AF triggering factors include (1) premature atrial contractions, (2) bradycardia, (3) sympathetic or parasympathetic stimulation, (4) atrial flutter and other supraventricular tachycardias, and (5) acute atrial stretch. Triggering foci of rapidly firing cells within the sleeves of atrial myocytes extending into the pulmonary veins have been identified as the underlying mechanism in up to 90% of paroxysmal AF cases. Other, less common sites of ectopic foci firing have been described in regions such as the superior vena cava, left atrial appendage, small muscle bundles in the ligament of Marshall, and the musculature of the coronary sinus [47,48]. Ectopic firing electrophysiology has been explained through three potential different mechanisms: (1) enhanced automaticity, (2) delayed afterdepolarizations (DADs), and (3) early afterdepolarizations (EADs). Atrial cell resting potential depends on the balance between the inward rectifying K^+^ current (IK1) and I funny current (If). Enhanced automaticity is caused by changes in this balance resulting from decreased IK1 and/or enhanced If current. DADs are caused by an abnormal diastolic release of Ca^2+^ from sarcoplasmic reticulum Ca^2+^ stores. When DADs become large enough to reach threshold potential, they cause cell firing, either as a single ectopic beat or as a sustained tachycardia. Ca^2+^-related triggered activity caused by abnormal Ca^2+^ handling is a strong candidate mechanism to underlie AF-generating ectopic foci. EADs involve abnormal secondary cell membrane depolarizations during repolarization phases; the prolongation of action potential duration (APD) is commonly associated with EADs, and it seems to be the pathophysiological basis of AF prevalence among long QT syndrome patients [49]. Several genetic mutations described in cases of familial AF directly alter channel proteins’ domain: for instance, mutations of *KCNE1* and *KCNQ1* affect potassium channels by a GOF effect, which accelerates repolarization and, hence, shortens the refractory period; LOF mutations of *KCNA5* (affecting Ikur potassium channels) cause the prolongation of the effective refractory period and enhanced propensity for EADs, which is exacerbated with sympathetic stimulation [12]; the *SCN5A* gene causes increased atrial peak sodium current, a potential trigger for increased atrial excitability [50]; and in vivo experiments showed also that *NPPA* mutations lead to a shortening of the monophasic APD as well as of the effective refractory period [51]. Although less common, other mechanisms unrelated to ion channel alterations have been described. These include changes in cell-to-cell electromechanical coupling, such as altered connexins and gap junction proteins, or an absent suppression of ectopic sinus node activity, as observed in *GATA* or *PITX2* mutations, respectively [24,25,26,52]. While these mechanisms help clarify the relationship between genetic variations and pathophysiology in familial AF, they are not necessarily shared by mechanisms initiating or maintaining AF in the general population.

## 4. Perpetuators of AF Maintenance

**Figure 2:**
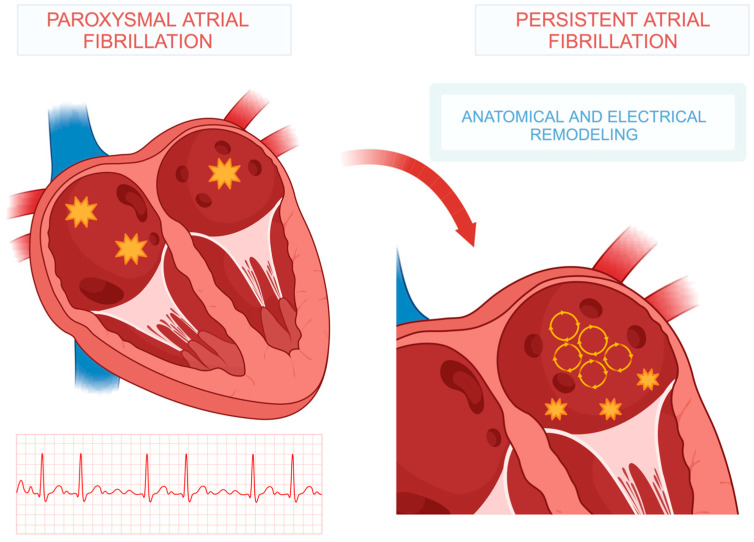
Schematic representation of AF progression process from paroxysmal to persistent.

AF can persist due to continuous firing from the initiating focus, often referred to as the “focal driver”. Although the focus itself fires regularly, it induces fibrillatory activity through wave breakup in portions of the atrium, where irregular, fractionated atrial responses prevent 1:1 conduction. Alternatively, AF may persist without focal drivers, resulting from a combination of electrical and structural remodeling processes promoting a chaotic disorganized electrical activity (Figure 2). This scenario often involves underlying functional or anatomical substrates. This explains why pulmonary vein isolation alone often fails to interrupt clinical arrhythmia in patients with persistent AF, regardless of the technique used for ablation and ablation transmurality [53,54,55,56]. Structural remodeling includes macroscopic changes, such as atrial dilatation and/or fibrosis [57]. Left atrial fibrosis and enlargement are frequently caused by underlying heart diseases, including valve disease and heart failure (with preserved or reduced ejection fraction), which explains the higher prevalence of AF in patients with these conditions [58]. Cardiovascular risk factors and comorbidities were key predictors of clinical AF progression.

Fibrotic myocardium exhibits slow and inhomogeneous conduction, with spatially nonuniform impulse propagation, likely due to reduced intercellular coupling and discontinuous architecture. These factors contribute to reentry circuits by slowing conduction velocity and by creating islands of non-excitable tissue around which reentry can occur. To maintain reentry, the impulse must traverse the circuit slowly enough for all points to regain excitability. If conduction time exceeds the longest functional refractory period in the circuit, reentry is sustained. Fibrosis facilitates this mechanism, explaining its role in AF progression [59]. Atrial dilation promotes AF by increasing circuit path space so that larger reentry circuits can be supported and/or a major number of smaller circuits can be maintained simultaneously. Not all reentries require an anatomical substrate to manifest. Local alterations of atrial tissue conduction properties can serve as a functional substrate for reentry to happen. These alterations can either precede AF onset or usually can be secondary to AF-induced remodeling (AF begets AF) [60].

**Figure 3:**
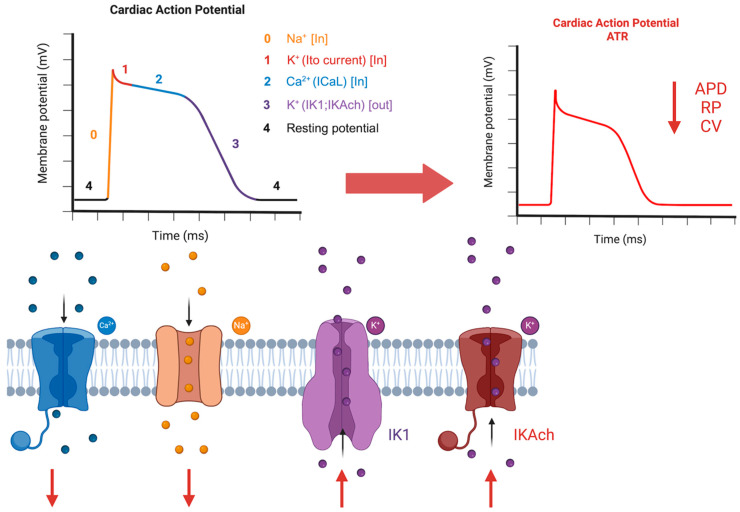
AF-mediated ion channel alterations. ATR is associated with (1) peak INa reduction due to Na^+^ channel downregulation, which leads to lower energy source and therefore lower conduction velocity; (2) lower ICaL during AP phase 2, which reduces APD, and (3) higher IK1 and IKAch and therefore lower APD and RP. ATR: atrial tachyarrhythmia remodeling; APD: action potential duration; CV: conduction velocity; RP: refractory period; INa: Na^+^ channel current; ICaL: L-type Ca^2+^ channel current; IK1: K^+^ inward rectifier channel current; IKAch: acetylcholine-regulated K^+^ channel current.

There is also evidence that atrial tachycardia remodeling (ATR), also known as electric remodeling, can alter atria conduction properties at several levels: ATR-mediated connexin changes (including decreased numbers, increased heterogeneity, and lateralization of connexins) contribute to decreasing conduction velocity by altering propagation between neighboring cells and increasing energy dispersion. Other mechanisms include reduced phase 0 Na current (INa) mediated by channel downregulation and deficient INa inactivation during the action potential plateau phase (this last mechanism described as cause of ADP and EADs in CHF patients) [61,62]. Moreover, ATR abbreviates atrial refractoriness by decreasing APD: primarily through L-type Ca current (ICaL) decrease but also via increased inward-rectifier K currents, such as the background current IK1 and a constitutively active form of acetylcholine-dependent K current (IKACh). Rapid atrial rates increase potentially cytotoxic Ca^2+^ loading. Autoprotective ICaL reductions occur via rapidly developing changes (ICaL inactivation) and more slowly developing changes in gene and protein expression [61,63] (Figure 3). Consistently, most of the previously described genes associated with familial AF and key variants contributing to AF risk encode proteins involved in these pathways. In fact, it is likely that genetic sequence alterations of channel proteins not only are relevant for AF initiation, but also all participate in promoting its maintenance: this is thought to be linked to their potential to further favoring the atrial tachycardia remodeling process, especially when compared to individuals not carrying the same mutations or genetic predisposition.

## 5. Conclusions

The genetics and pathophysiology of AF are not yet fully understood. However, experimental studies have begun to uncover potential mechanisms involved in AF initiation and maintenance. Moreover, several genetic alterations identified in both the general AF population and familial cases of early-onset AF highlight the significant role of genetic predisposition in AF triggers and drivers. In early-onset AF, genetic factors appear to have a greater influence than environmental or acquired factors, whereas the opposite is observed in the late-onset form of the disease. Understanding the complex interplay of these contributors will be crucial in the coming years for developing more personalized and tailored strategies for AF prevention and management.

## Figures and Tables

**Table 2:** A list of sentinel variants linked to AF from latest GWAS publications [35,36,37]. To date, there are at least 138 AF loci identified in single-variant testing with *p* < 5 × 10^−8^. * The listed genes are either the nearest gene(s) or the egene. Rsid: reference SNP id. An eGene is defined as a gene with an eQTL to the variant at an AF GWAS locus. Adapted from Roselli et al. [38].

Rsid	Nearest Gene(s) or eGene *	Rsid	Nearest Gene(s) or eGene *	Rsid	Nearest Gene(s) or eGene *
rs187585530	*UBE4B*	rs716845	*KCNN2*	rs1822273	*NAV2*
rs880315	*CASZI*	rs2012809	*FBN2*, *SLC27A6*	rs949078	*SORL1*, *MIR100HG*
rs7529220	*HSPG2*, *CELA3B*	rs34750263	*WNT8A*, *NME5*	rs76097649	*KCNJ5*
rs2885697	*SCMH1* *	rs174048	*ARHGAP26*, *NR3C1*	rs6490029	*CUX2*
rs11590635	*AGBL4*	rs12188351	*SLIT3*	rs10842383	*LINC00477*, *BCAT1*
rs56202902	*FAF1*	rs6882776	*NKX2-5*	rs113819537	*SSPN* *
rs146518726	*Clorf185*	rs73366713	*ATXN1*	rs12809354	*PKP2*
rs12044963	*KCND3*	rs34969716	*KDM1B*	rs7978685	*NACA*
rs4484922	*CASQ2* *	rs1307274	*C6orf1*, *NUDT3*	rs35349325	*BEST3*
rs79187193	*GJA5*	rs3176326	*CDKN1A*	rs11180703	*KRR1*, *PHLDAI*
rs11264280	*KCNN3*, *PMVK*	rs6907805	*CGA*, *ZNF292*	rs883079	*TBX5*
rs72700114	*METTL11B*, *LINC01142*	rs210632	*GOPC*	rs12810346	*TBX5-AS1*, *TBX3*
rs608930	*GORAB*, *PRRX1*	rs17079881	*SLC35F1*	rs10773657	*HIPIR*
rs10753933	*PPFIA4* *	rs13191450	*GJA1*, *HSF2*	rs12298484	*DNAH10*
rs4951261	*NUCKSI*	rs12208899	*LINC00326*, *EYA4*	rs6560886	*FBRSLI*
rs6546620	*KIF3C*	rs117984853	*UST*	rs9580438	*LINC00540*, *BASP1P1*
rs6742276	*XPO1*	rs11768850	*SUNI*	rs35569628	*CUL4A*
rs2540949	*CEP68* *	rs55734480	*DGKB*	rs28631169	*MYH7*
rs10165883	*SNRNP27*	rs6462078	*CREB5*	rs2145587	*AKAP6*
rs72926475	*REEP1*, *KDM3A*	rs74910854	*PMS2P2* *	rs73241997	*SNX6*, *CFL2*
rs28387148	*GYPC* *	rs11773884	*CDK6*	rs2738413	*SYNE2*
rs67969609	*TEX41*	rs62483627	*COG5*	rs74884082	*DPF3*
rs12992412	*MBD5*	rs11773845	*CAV1*	rs10873299	*LRRC74*, *IRF2BPL*
rs56181519	*WIPF1* *	rs55985730	*OPNISW*	rs147301839	*MYZAP*
rs2288327	*FKBP7* *	rs7789146	*KCNH2*	rs62011291	*USP3*
rs3820888	*SPATS2L* *	rs35620480	*LINC00208*, *GATA4*	rs12591736	*TLE3*, *UACA*
rs35544454	*ERBB4*	rs7508	*ASAH1* *	rs74022964	*HCN4*, *REC114*
rs6810325	*MKRN2* *	rs7846485	*XPO7*	rs12908004	*LINC00927*, *ARNT2*
rs73032363	*THRB*	rs62521286	*FBXO32*	rs12908437	*IGF1R* *
rs6790396	*SCN10A*	rs35006907	*MTSSI*, *LINC00964*	rs2286466	*RPL3L* *
rs34080181	*SLC25A26* *	rs7460121	*MIR30B*	rs2359171	*ZFHX3*
rs17005647	*FRMD4B*	rs6993266	*PTK2*	rs7225165	*YWHAE*, *CRK*
rs7632427	*EPHA3*	rs4977397	*SLC24A2*, *MLLT3*	rs8073937	*POLR2A*, *TNFSF12*
rs17490701	*PHLDB2*	rs4385527	*C9orf3*	rs72811294	*MYOCD*
rs1278493	*PPP2R3A*	rs4743034	*ZNF462*	rs11658278	*ZPBP2*
rs4855075	*GNB4*	rs10760361	*PSMB7*	rs242557	*MAPT*
rs60902112	*XXYLT1*	rs2274115	*LHX3*	rs76774446	*GOSR2*
rs9872035	*PAK2*	rs2296610	*NEBL*	rs7219869	*KCNJ2*, *CASC17*
rs3822259	*WDR1*	rs7919685	*NRBF2* *	rs12604076	*CYTH1*
rs1458038	*PRDM8*, *FGF5*	rs7096385	*SIRTI*	rs9953366	*SMAD7*
rs3960788	*UBE2D3* *	rs60212594	*SYNPO2L*	rs8088085	*MEX3C*
rs2129977	*PITX2*, *C4orf32*	rs11001667	*C10orf11*	rs2145274	*CASC20*, *BMP2*
rs55754224	*CAMK2D*	rs1044258	*C10orf76*	rs7269123	*C20orf166*
rs10213171	*ARHGAP10*	rs11598047	*NEURL*	rs2834618	*LOC100506385*
rs10520260	*HAND2-AS1* *	rs2047036	*SH3PXD2A*	rs465276	*TUBA8*
rs6596717	*LOC102467213*, *EFNA5*	rs10749053	*RBM20*	rs133902	*MYO18B*
